# Efficacy and safety of apatinib combined with transarterial chemoembolization for hepatocellular carcinoma with portal venous tumor thrombus: a retrospective study

**DOI:** 10.18632/oncotarget.20140

**Published:** 2017-08-10

**Authors:** Changfu Liu, Wenge Xing, Tongguo Si, Haipeng Yu, Zhi Guo

**Affiliations:** ^1^ Department of Interventional Treatment, Tianjin Medical University Cancer Institute and Hospital, Tianjin, China; ^2^ National Clinical Research Center for Cancer, Tianjin, China; ^3^ Key Laboratory of Cancer Prevention and Therapy, Tianjin, Tianjin, China; ^4^ Tianjin’s Clinical Research Center for Cancer, Tianjin, China

**Keywords:** hepatocellular carcinoma, portal vein tumor thrombosis, apatinib, transarterial chemoembolization, efficacy

## Abstract

**Objective:**

To investigate the efficacy and safety of combined therapy with apatinib and transarterial chemoembolization (TACE) for hepatocellular carcinoma with portal venous tumor thrombus (PVTT).

**Materials and Methods:**

We retrospectively analyzed 19 patients with hepatocellular carcinoma with PVTT who were treated with apatinib and TACE at a single center between January 2015 and January 2017. Clinical information on the patients was collected. Adverse events, overall survival, progression-free survival, objective response rate, and disease-control rate based on mRECIST criteria (American Association for the Study of Liver Diseases, 2008) were reviewed and evaluated.

**Results:**

All patients had complete follow-up records and the median follow-up time was 13 months (1–24 months). Among the 19 patients, 63.16% achieved a partial response and 21.05% achieved stable disease. The objective response and disease-control rates for the tumor were 63.16% and 84.21%, respectively, and the objective response and disease-control rates for PVTT were 10.93% and 89.47%, respectively. The median overall survival was 11.9 months, and the 6-month and 1-year overall survival rates were 94.7% and 48.8%, respectively. The median progression-free survival rate was 8.1 months, and the 6-month and 1-year rates were 73.3% and 22.9%, respectively. The most common apatinib-related adverse events were hand-foot-skin reaction, fatigue, dyspepsia, diarrhea, and hypertension, and the most common TACE-related adverse event was fever. No procedure-related mortality or grade 4 adverse events were observed, but grade 3 adverse events were observed in two patients.

**Conclusions:**

This exploratory study suggested that apatinib combined with TACE treatment was safe and might improve overall and progression-free survival in patients with hepatocellular carcinoma with PVTT. Further randomized controlled trials are needed to clarify the potential role of apatinib in hepatocellular carcinoma with PVTT.

## INTRODUCTION

Hepatocellular carcinoma is the fifth most common malignancy and the third leading cause of cancer-related deaths worldwide [1, 2]. Early diagnosis of liver cancer is improving thanks to the increased use of imaging techniques such as ultrasonography and computed tomography (CT), and tumor-related mortality is decreasing. However, 70%–80% of hepatocellular carcinoma cases in China are diagnosed at an advanced stage. Furthermore, portal venous tumor thrombus (PVTT) is observed in 12.5%–62.2% of patients with advanced hepatocellular carcinoma at their initial visit [3, 4]. PVTT may cause extensive intrahepatic dissemination of the tumor through the portal tract, decrease blood supply to the normal liver, and cause portal hypertension resulting in the rupture of collateral vessels, ascites, hepatic encephalopathy, and deteriorating liver function [5, 6]. These problems will affect the choice of treatments for patients with hepatocellular carcinoma.

Standard treatments for hepatocellular carcinoma include radical resection, liver transplantation, and percutaneous ablation, with associated 5-year survival rates of 37%–70% [7–9]. However, complete resection is not feasible in most hepatocellular carcinoma patients with PVTT, and the prognosis for these patients is poor, with a median overall survival of 2.7–4 months if left untreated [4, 10, 11]. Transarterial chemoembolization (TACE) is generally accepted as an effective palliative treatment for patients with unresectable hepatocellular carcinoma. However, although some studies have suggested that it may also be safe for selected patients with PVTT, its efficacy in these patients has remained unsatisfactory [12] and other treatment options, such as systemic or regional chemotherapy, adoptive immunotherapy, and intra-arterial radioiodine injection, have shown no survival benefit [13, 14]. Improved multi-disciplinary consultation models and increased use of combination therapy have been applied in patients with hepatocellular carcinoma with PVTT, including TACE combined with three-dimensional conformal radiotherapy or with sorafenib, which have demonstrated efficacy in terms of local disease control, symptomatic relief, and increased survival [6, 11, 15].

Sorafenib is an oral multi-kinase inhibitor with anti-proliferative and antiangiogenic effects, which is recommended as the first-line option for advanced stage hepatocellular carcinoma according to the Barcelona Clinic Liver Cancer (BCLC) staging system [14, 16, 17]. The results of two clinical studies have shown that sorafenib significantly prolonged overall survival, delayed disease progression, and was well-tolerated in patients with advanced hepatocellular carcinoma [18, 19]. However, the survival advantages and treatment responses were modest; for patients in the Asia–Pacific region, the median overall survival rates of patients treated with sorafenib and placebo were 6.5 and 4.2 months, respectively, and the objective response rate was only 3.3% [18]. In addition, the high cost of sorafenib limits its application for advanced hepatocellular carcinoma in China.

Apatinib is a novel receptor tyrosine kinase inhibitor that selectively targets vascular endothelial growth factor (VEGF) receptor 2, with a binding affinity 10 times that of sorafenib [20]. Apatinib significantly reduced tumor growth in several established human tumor xenograft models by inhibiting tumor-induced angiogenesis [21], and exhibited antitumor activity in clinical trials in patients with gastric cancer [22–25]. Apatinib has also shown promising therapeutic effects against diverse tumor types, including gastric cancer, ovarian cancer, breast cancer, and hepatocellular carcinoma, in several phase II clinical trials [20, 23, 26–28]. Furthermore, the low price of apatinib facilitates its application in China.

The present study was designed to assess the safety and survival benefit of apatinib combined with TACE in hepatocellular carcinoma patients with PVTT.

## RESULTS

### Patients and treatment

Nineteen patients with hepatocellular carcinoma with PVTT were enrolled in this study. All the patients were newly diagnosed and had not received any antineoplastic therapy. There were 14 men and five women, with a median age of 62 years (range 48–72 years). All the enrolled patients had pathologically confirmed hepatocellular carcinoma BCLC stage C. Distant metastases were observed in the lymph nodes (*n =* 9, 47.37%), lungs (*n =* 2, 10.53%), and bone (*n =* 1, 5.23%). Ten patients (52.63%) had nodular-type hepatocellular carcinoma, and nine patients (47.37%) had diffuse-type hepatocellular carcinoma, classified according to the Liver Cancer Study Group of Japan criteria, as described previously [9, 16]. Six (31.58%), 10 (52.63%), and three (15.79%) patients had types Vp2, Vp3, and Vp4, respectively. All patients were treated with apatinib in combination with TACE during the study period. The baseline characteristics of the enrolled patients are shown in Table 1.

**Table 1:** The baseline characteristics of enrolled patients and tumor response 1 month after combination treatment

NO.	Gender	Age	Pathology	BCLC stage	Metastasis site	VEGF	Type of PVTT	Gross morphological type	Response
**1**	M	62	HCC	C	-	+	Vp2	Nodular	PR
**2**	M	65	HCC	C	Lymph nodes	+	Vp2	Diffuse	PR
**3**	M	56	HCC	C	Lung	+	Vp3	Diffuse	PD
**4**	M	48	HCC	C	Lymph nodes	+	Vp4	Nodular	PR
**5**	F	55	HCC	C	-	+	Vp2	Nodular	PR
**6**	M	57	HCC	C	Lymph nodes	N/A	Vp3	Nodular	SD
**7**	M	72	HCC	C	-	-	Vp3	Diffuse	PR
**8**	F	59	HCC	C	Lymph nodes	+	Vp3	Nodular	PD
**9**	M	67	HCC	C	Lung	+	Vp2	Diffuse	PR
**10**	M	66	HCC	C	-	+	Vp3	Diffuse	SD
**11**	M	67	HCC	C	Lymph nodes	-	Vp4	Nodular	PR
**12**	M	64	HCC	C	Bone	+	Vp2	Nodular	PD
**13**	M	63	HCC	C	-	N/A	Vp3	Nodular	PR
**14**	F	54	HCC	C	N/A	-	Vp3	Diffuse	PR
**15**	F	57	HCC	C	Lymph nodes	-	Vp3	Diffuse	PR
**16**	F	52	HCC	C	-	+	Vp3	Diffuse	SD
**17**	M	62	HCC	C	Lymph nodes	+	Vp4	Diffuse	PR
**18**	M	65	HCC	C	Lymph nodes	+	Vp3	Nodular	PR
**19**	M	53	HCC	C	Lymph nodes	-	Vp2	Nodular	SD

VEGF: vascular endothelial growth factor; PR: partial response; SD: stable disease; PD: progressive disease; N/A: not available; BCLC: Barcelona Clinic Liver Cancer; PVTT: portal venous tumor thrombus; HCC: hepatocellular carcinoma; M: male; F: female.

### Efficacy

#### Overall and PVTT response rates

**Figure 1:**
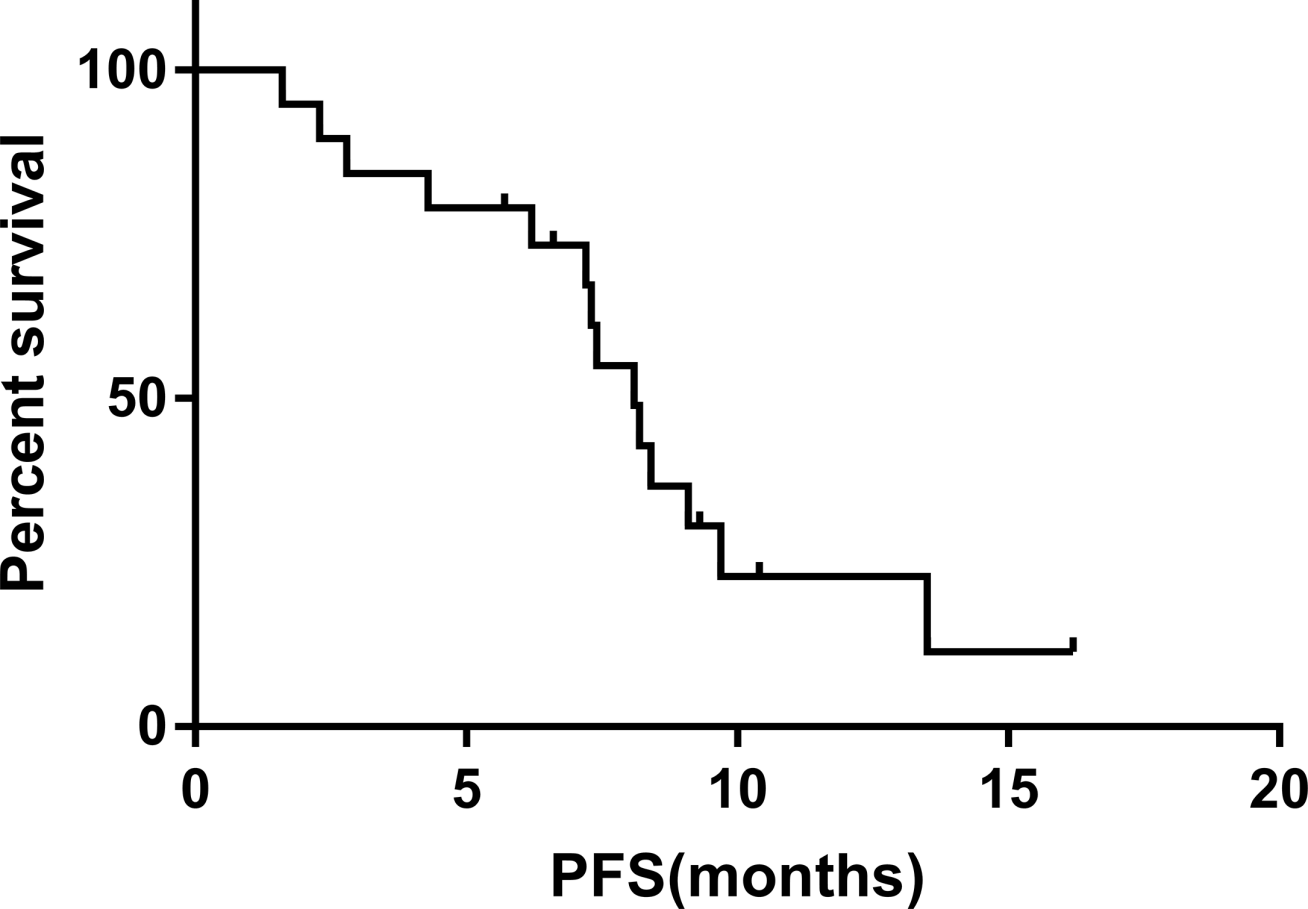
Progression-free survival in patients with advanced hepatocellular carcinoma and PVTT treated with combined apatinib and TACE The median progression-free survival was 8.1 months, with 6-month and 1-year rates of 73.3% and 22.9%, respectively.

**Figure 2:**
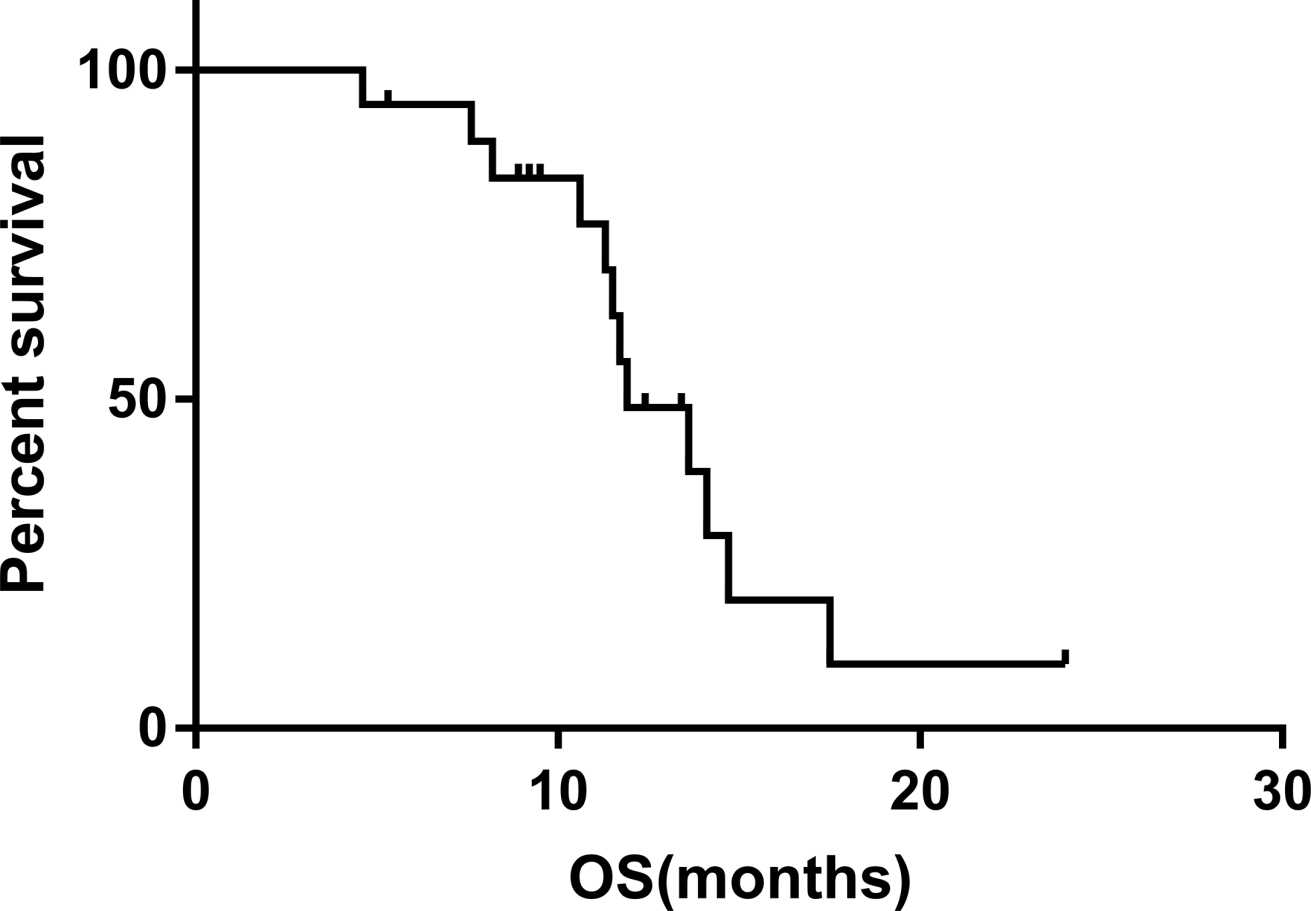
Overall survival in patients with advanced hepatocellular carcinoma and PVTT treated with combined apatinib and TACE The median overall survival was 11.9 months, with 6-month and 1-year rates of 94.7% and 48.8%, respectively.

**Figure 3:**
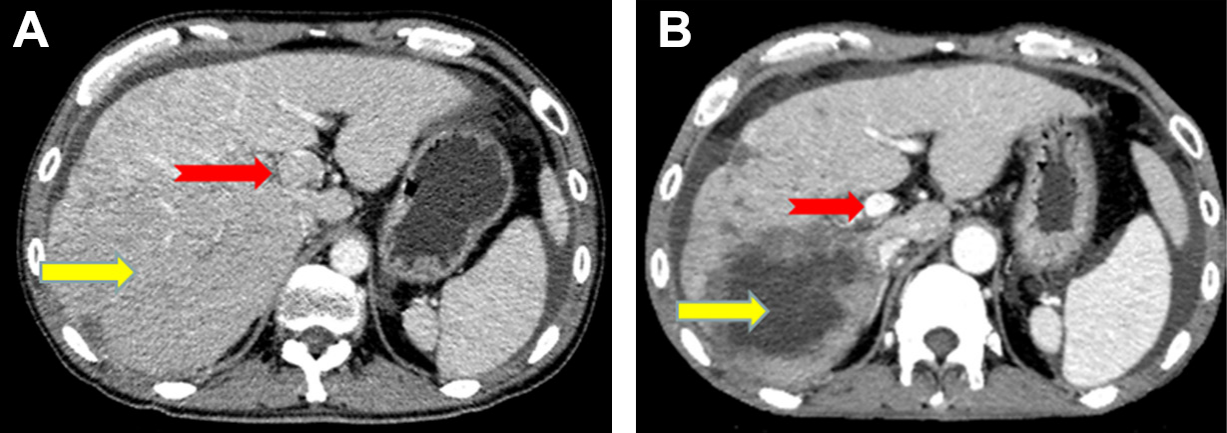
Image of a 48-year-old man with hepatocellular carcinoma and PVTT who showed a partial response after combined apatinib and TACE treatment Contrast-enhanced CT at diagnosis showed a 134 mm diameter hepatocellular carcinoma nodule (yellow arrow) and multiple small metastatic lesions located in the liver, together with PVTT in the left and main portal vein (red arrow). CT images 1 month after diagnosis showed intrahepatic lesions in numerous non-enhanced areas (yellow arrow) and almost complete absence of PVTT without definite enhancement (red arrow).

All patients had complete follow-up records and all the responses were therefore evaluable. Tumor response was assessed using mRECIST criteria. Treatment efficacy was evaluated 1 month after the start of treatment. The overall objective response and disease-control rates of the combined treatment were 63.15% and 84.21%, respectively, including 12 partial responses, four cases of stable disease, and three cases of progressive disease. The median progression-free survival was 8.1 months (Figure 1) and the median overall survival was 11.9 months (Figure 2). In terms of PVTT response rates, two patients (10.53%) achieved partial responses, 15 patients (78.94%) had stable disease, and two patients (10.53%) had progressive disease 1 month after therapy. The objective response rate of PVTT was 10.53% and the disease-control rate was 89.47%. Examples of follow-up CT images in patients who achieved a partial response 1 month after combined apatinib with TACE therapy are shown in Figure 3.

### Correlations among D-dimer, AFP, lesion diameter, and PVTT diameter

**Figure 4:**
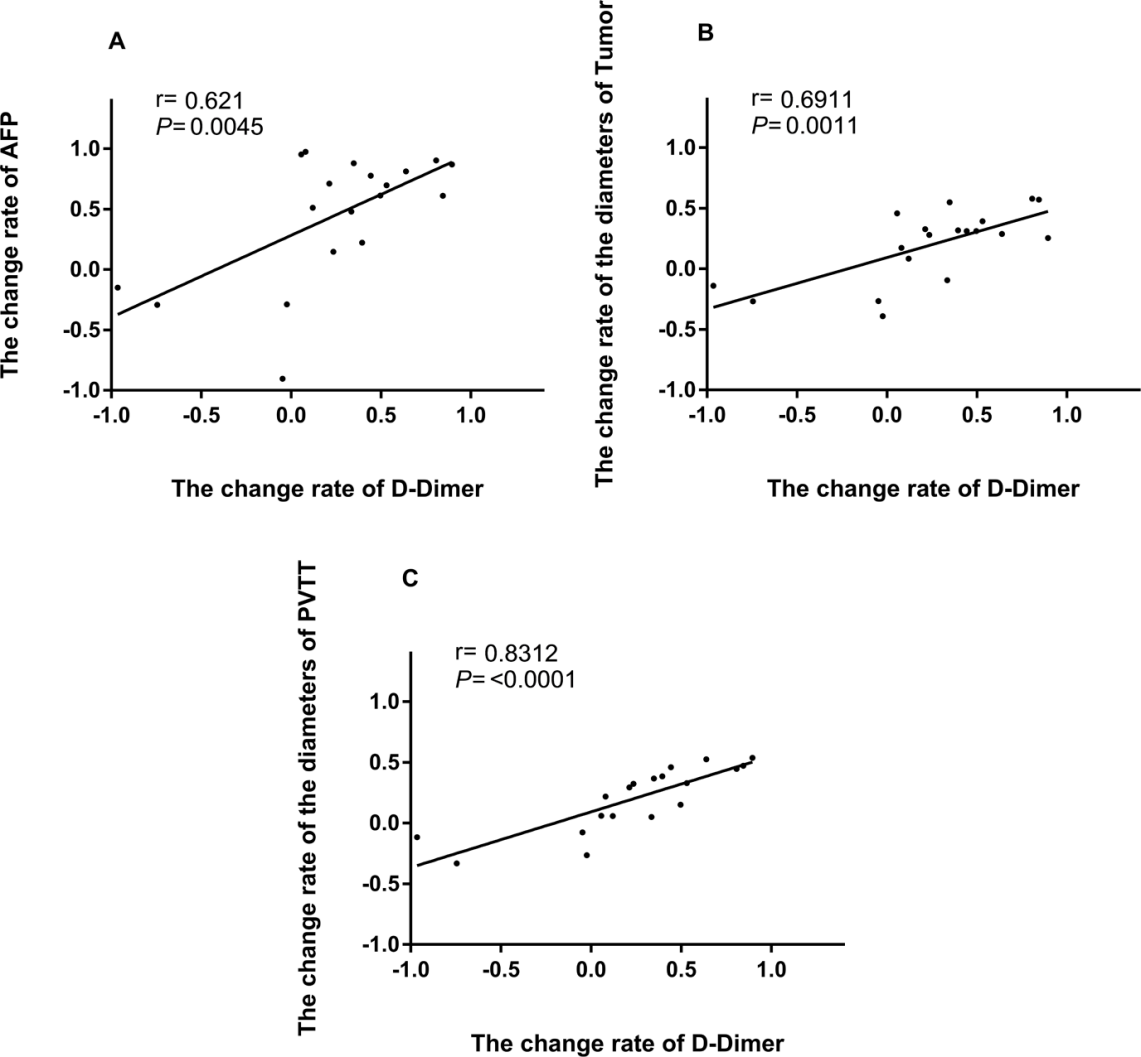
Association between the rate of change of D-dimer and rates of change of AFP, tumor diameter, and PVTT diameter (**A**) Relationship between the rates of change of D-dimer and AFP after 1 month of apatinib combined with TACE treatment. (**B**) Relationship between the rates of change of D-dimer and tumor diameter after 1 month of apatinib combined with TACE treatment. (**C**) Relationship between the rates of change rate of D-dimer and PVTT after 1 month of apatinib combined with TACE treatment.

Decreases in D-dimer, AFP, lesion diameter, and PVTT diameter were calculated 1 month after treatment with apatinib combined with TACE, and the relationships among the four indicators were analyzed (Figure 4). Changes in D-dimer were positively correlated with AFP, lesion diameter, and PVTT diameter after 1 month of therapy (*P* = 0.036, *P* < 0.001, and *P* = 0.003, respectively) (Figure 4A–4C).

### Safety

#### Effects of combination therapy on liver and kidney functions

There were no significant differences between preoperative and postoperative liver function, renal function, coagulation function, hepatitis B virus (HBV) DNA levels, and Eastern Cooperative Oncology Group (ECOG) criteria (*P* > 0.05). Regular anti-viral treatment was initiated in patients with HBV DNA replication. Liver damage due to combined treatment was slight and acceptable (Table 2).

**Table 2:** The routine laboratory tests of enrolled patients before and after treatment ($\bar{\text{x}}$ ± s)

	Prior treatment	Posttreatment	*t*	*P*
ALT (U/L)	44.74 ± 28.83	41.05 ± 16.49	1.01	0.372
AST (U/L)	48.95 ± 30.89	54.59 ± 16.10	−1.47	0.159
TBIL (μmol/L)	20.75 ± 7.16	18.99 ± 5.45	2.08	0.052
DBIL(μmol/L)	3.51 ± 0.81	3.17 ± 0.47	1.73	0.100
ALB(g/L)	38.80 ± 4.55	38.99 ± 1.93	−0.21	0.840
INR(INR)	11.61 ± 0.89	11.49 ± 1.47	0.35	0.731
Cr (μmol/L)	63.74 ± 17.68	66.53 ± 15.44	−1.09	0.289
UREA(mmol/L)	5.26 ± 1.98	5.01 ± 0.96	0.72	0.479
HBV-DNA(IU/ml)	21932.32 ± 53647.23	173.15 ± 279.32	1.77	0.093
ECOG	0.36 ± 0.50	0.47 ± 0.51	−0.81	0.429

ALT: alanine aminotransferase; AST: aspartate transaminase; TBIL: total bilirubin; DBIL: conjugated bilirubin; ALB: Serum albumin; INR: International Normalized Ratio; Cr: creatinine; HBV: hepatitis B virus; ECOG: Eastern Cooperative Oncology Group.

### Adverse effects of combination therapy

Treatment-related adverse events included hand-foot-skin reaction (*n =* 17, 89.47%), hypertension (*n =* 15, 78.95%), fatigue (*n =* 12, 63.15%), diarrhea (*n =* 11, 57.89%), anorexia (*n =* 9, 47.37%), dyspepsia (*n =* 6, 31.58%), proteinuria (*n =* 6, 31.58%), thrombocytopenia (*n =* 5, 26.32%), nausea (*n =* 4, 21.05%), oral mucositis (*n =* 3, 15.79%), headache/dizziness (*n =* 3, 15.79%), hypoproteinemia (*n =* 2, 10.53%), pharyngolaryngeal pain (*n =* 1, 5.26%), elevated transaminases (*n =* 1, 5.26%), hyperbilirubinemia (*n =* 1, 5.26%), and hoarseness (*n =* 1, 5.26%). Most adverse events were grade I or II. The reported grade 3 drug-related adverse events were hand-foot-skin reaction in one patient (5.26%) and diarrhea in one patient (5.26%). No grade 4 adverse events were observed. All the adverse events could be relieved by and tolerated after drug treatment or dose reduction, and all patients continued to take the study drugs and were followed up. The adverse events related to apatinib are shown in Table 3. The most common adverse events related to TACE were fever (*n =* 9, 47.37%), mild epigastric pain (*n =* 7, 36.84%), and nausea (*n =* 4, 21.05%), all of which were relieved during the first week with appropriate treatment.

**Table 3:** Adverse events

Adverse events (%)	All grades *n* (%)	Grade ≥ 3 *n* (%)
Fatigue	12 (63.15)	0 (0)
Headache/Dizzy	3 (15.79)	0 (0)
Diarrhea	11 (57.89)	1 (5.26)
Anorexia	9 (47.37)	0 (0)
Vomit	0 (0)	0 (0)
Stomachache	0 (0)	0 (0)
Nausea	4 (21.05)	0 (0)
Alimentary tract hemorrhage	0 (0)	0 (0)
Dysphagia	0 (0)	0 (0)
Pharyngolaryngeal pain	1 (5.26)	0 (0)
Dyspepsia	6 (31.58)	0 (0)
Mucositis oral	3 (15.79)	0 (0)
Dysgensia	0 (0)	0 (0)
HFSR	17 (89.47)	1 (5.26)
Hypertension	15 (78.95)	0 (0)
Proteinuria	6 (31.58)	0 (0)
Elevated transaminase	1 (5.26)	0 (0)
Hyperbilirubinemia	1 (5.26)	0 (0)
Elevated GGT	0 (0)	0 (0)
Alkaline phosphatase	0 (0)	0 (0)
Hypoproteinemia	2 (10.53)	0 (0)
Leukopenia	0 (0)	0 (0)
Neutropenia	0 (0)	0 (0)
Aglobulism	0 (0)	0 (0)
Thrombocytopenia	5 (26.32)	0 (0)
Hoarseness	1 (5.26)	0 (0)

HFSR: hand-foot-skin reaction; GGT: gamma-glutamyl transpeptidase.

## DISCUSSION

PVTT occurs in a substantial proportion of hepatocellular carcinoma patients and is a poor prognostic factor in 20%–60% of cases [29–31]. Hepatocellular carcinoma combined with PVTT is often associated with a high degree of malignancy and treatment difficulties, leading to hepatic function damage, portal hypertension, and other risks, and a maximum survival time of 3 months if left untreated. The recommended treatment for advanced hepatocellular carcinoma according to BCLC is sorafenib [8]. However, the efficacy of sorafenib for hepatocellular carcinoma with PVTT is limited, and two randomized controlled trials found response rates of no more than 4% and 2%–3.3%, respectively [18, 19]. Furthermore, a series of clinical trials of sorafenib combined with TACE for the treatment of hepatocellular carcinoma with PVTT showed inconsistent efficacy. Some found no advantage of combination therapy over sorafenib monotherapy[32], while others found that an interrupted therapeutic scheme of TACE plus sorafenib was safe, and might improve overall survival in hepatocellular carcinoma patients with PVTT [11]. Furthermore, sorafenib is too expensive for most patients in China to afford. It is therefore necessary to conduct new clinical trials in patients with advanced hepatocellular carcinoma in China, to identify more efficient and suitable treatments for Chinese patients.

Recent exploratory studies of apatinib in a variety of tumors suggested that it was efficient, with moderate adverse events [33–35]. However, to the best of our knowledge, no studies have assessed the outcomes in hepatocellular carcinoma patients with PVTT treated with apatinib combined with TACE. We therefore conducted the current clinical study to verify the feasibility of this therapeutic regimen. The results demonstrated that apatinib plus TACE was effective in patients with advanced hepatocellular carcinoma with PVTT, with median progression-free and overall survival rates of 8.1 and 11.9 months, respectively. This overall survival is longer than that reported for either sorafenib or TACE alone (5.6–8.1 and 3.8–9.5 months, respectively) and similar to that for sorafenib combined with TACE [11]. The effectiveness of apatinib combined with TACE may be attributed to the integrated control effect of locoregional plus systemic therapy. The combination therapy may cause superselective hepatic arterial embolization without affecting the blood supply to the normal liver, thus avoiding further liver ischemic damage. Furthermore, TACE induces tumor hypoxia and promotes VEGF expression, while apatinib can inhibit the VEGF receptor and VEGF signaling, which are important for tumor recrudescence. The diameters of the portal vein and hepatic artery were decreased after treatment with apatinib combined with TACE, thus increasing portal vein pressure and reducing the blood supply to the liver. There was no significant change in the hepatic artery or portal vein in patients enrolled in the current study, and no increased gastrointestinal bleeding or ascites.

Previous trials showed objective response and disease-control rates of sorafenib combined with TACE of 19.5% and 80.5%, respectively [11], while two randomized controlled trials showed rates for sorafenib alone of 2%–3% and 57.3%–73%, respectively [18, 19]. The equivalent objective response and disease-control rates of apatinib combined with TACE were 63.16% and 84.21%, which appear to be higher than those for sorafenib combined with TACE or sorafenib alone. There are several possible reasons for these differences, including the current evaluation of the tumor-curative effect according to the mRECIST criteria, which is more suitable for the local treatment of liver cancer or molecular targeted therapy. Another possible reason is that apatinib is a receptor tyrosine kinase inhibitor that selectively targets VEGF receptor 2, with a binding affinity 10 times higher than sorafenib. Moreover, all the enrolled patients were Child–Pugh score A, which is a known predictor of survival in patients with hepatocellular carcinoma. Furthermore, the number of enrolled patients in the current study was limited. The objective response and disease-control rates of PVTT were 10.93% and 89.47%, respectively. This objective response rate of PVTT was lower than that of radiotherapy combined with TACE, but the disease-control rate was similar [6]. This may be because three-dimensional conformal radiotherapy and image-guided radiotherapy cause direct necrosis of the PVTT by delivering a higher dose [36–38]. These results suggest that apatinib combined with TACE is an effective treatment modality in terms of both response and disease control.

The relationship between activation of the hemostatic system and tumors has received much attention [39–41]. Previous studies have shown that malignant tumors can activate the coagulation and fibrinolytic system, and tumor-induced coagulation and fibrin formation are required for tumor angiogenesis, metastasis, and invasion. Cross-linked fibrin is a crucial source of bio-available fibrin for tumor cells in the vasculature, and may provide a stable framework for endothelial cell and tumor cell migration during angiogenesis and invasion [41, 42]. D-dimer, a degradation product of cross-linked fibrin, is the most frequently activated indicator of the coagulation and fibrinolytic system and has been associated with chemoresistance and poor disease outcome in many different forms of cancer. We performed a linear correlation analysis and showed that D-dimer levels were positively correlated with the rates of change in tumor diameter, PVTT diameter, and AFP. D-dimer thus appears to be a sensitive indicator of tumor prognosis and for determining the therapeutic effects of apatinib combined with TACE in hepatocellular carcinoma patients with PVTT.

The results of this study found no significant difference in liver or kidney function following treatment. In terms of HBV, combination therapy did not increase HBV DNA levels or activation of hepatitis in HBV DNA-negative patients, and entecavir inhibited HBV replication in patients with high virus titers prior to combination therapy. Careful monitoring of HBV DNA levels and antiviral therapy should thus be considered to reduce the risk of HBV infection reactivation in patients receiving combination therapy.

Apatinib has many potential adverse reactions, similar to other molecular targeted drugs, though no serious adverse events were observed in the current study. The most frequent adverse events were hand-foot-skin reaction, hypertension, fatigue, diarrhea, and anorexia, which are common adverse events of all tyrosine kinase inhibitors. Other adverse events including dyspepsia, proteinuria, thrombocytopenia, nausea, and oral mucositis were less frequent, but contributed to patient discomfort. Most adverse events in the current study were grade 1 or 2 and were well-tolerated by patients without the need for dose reduction or suspension of medication. These symptoms were gradually alleviated and disappeared within 1 or 2 weeks. Grade 3 adverse events were reduced to grade 1 after drug discontinuation or dose reduction to 250mg. Our results also suggested that the incidence of adverse events may be related to drug efficacy.

There were some limitations to the current study. First, it was a retrospective, single-arm, single-center study with no control group. Second, the number of patients enrolled in the study was small. Third, all the patients had Child–Pugh class A liver function, indicating good liver functional reserve and no serious damage, which can directly affect treatment choice and prognosis. Fourth, the quality of life of the patients before treatment ranged from 0 to 1, as evaluated by ECOG score. Moreover, tumor response was evaluated by mRECIST criteria, which are more suitable for molecular targeted therapy and local treatment of liver cancer.

In conclusion, the combination of apatinib and TACE was safe and effective as an initial treatment modality for hepatocellular carcinoma patients with PVTT, with only mild impacts on liver function, renal function, and HBV replication. Moreover, the side effects of apatinib combined with TACE were no more severe than those of sorafenib combined with TACE. Importantly, apatinib is cheaper than other molecular targeted agents and is thus more suitable for use in Chinese patients with hepatocellular carcinoma. Apatinib combined with TACE might thus represent an alternative treatment modality for patients with advanced hepatocellular carcinoma with PVTT. Further randomized controlled trials are needed to confirm these results.

## MATERIALS AND METHODS

### Patients and treatment

Approval for this study was obtained from the institutional ethics committee, and written informed consent was obtained from each patient before the procedure. We retrospectively reviewed the medical records of patients who underwent TACE combined with apatinib therapy between January 2015 and January 2017 at Tianjin Medical University Cancer Hospital and Institute. All patients were screened for eligibility according to the following inclusion criteria [10, 11, 43–45]: (1) newly diagnosed hepatocellular carcinoma BCLC C with PVTT; (2) pathological biopsy diagnosis of hepatocellular carcinoma and contrast-enhanced CT or magnetic resonance imaging (MRI) scan obtained; (3) Child–Pugh class A; (4) ECOG performance status score of 0 to 1; (5) adequate cardiopulmonary, hepatic, and renal functions; (6) absence of infection; and (7) life expectancy > 3 months. The exclusion criteria were coagulation dysfunction, international normalized ratio > 1.7 or platelets < 50 × 10^3^, and prior therapy with a tyrosine kinase inhibitor or TACE. All the patients enrolled in this study were treated with TACE. The embolization and chemotherapeutic agents were kelp microgel beads (Beijing ShengYiYao Technology and Development Co., Ltd., Beijing, China) and epirubicin (Pfizer, New York, NY, USA), respectively. Depending on the size, location, and arterial supply of the tumor, the tip of the catheter was advanced into the tumor-feeding branches and embolization was performed using 100–300 μm or 150–450 μm diameter microgel beads, followed by a final infusion of 40 mg of epirubicin. Patients were then commenced on apatinib (Jiangsu Hengrui Medicine, Lianyungang, China) at a dose of 500 mg once a day, reduced to 250 mg in the event of intolerable side effects. A treatment cycle was defined as 1 month. Contrast-enhanced CT or MRI was performed 1 month after the treatment to assess treatment efficacy. If residual viable tumors were confirmed, TACE was repeated in patients who met the eligibility criteria. A 3-day interruption in apatinib was adopted after each subsequent TACE cycle.

### Efficacy and safety assessments

Tumor responses were evaluated by evaluators who were blinded to the patients’ diagnoses, using mRECIST criteria. mRECIST requires evaluation of only those areas of the tumor showing arterial enhancement on contrast-enhanced CT or dynamic MRI [14]. The target lesions in each case were defined by two interventional radiologists after review of the contrast-enhanced CT and/or dynamic MRI images.

PVTT response was evaluated by serial CT scans performed 1 month after therapy. The product of the largest perpendicular diameter of the tumor thrombus was calculated and compared with the initial value. Complete response was defined as the complete disappearance of the PVTT, partial response as a ≥ 50% decrease in thrombus diameter, stable disease as a < 50% decrease or < 25% increase in thrombus diameter, and progressive disease as a ≥ 25% increase in thrombus diameter.

Tumor responses were evaluated as objective response (complete response plus partial response). The objective response rate was the percentage of patients with an objective response among all cases, and the disease-control rate was the percentage of patients with complete or partial response, or stable disease. Progression-free survival was based on the length of time from initial treatment until disease progression. Overall survival was based on the length of time from initial treatment until time of death, or final hospital visit in patients who remained alive at the end of the observation period.

AFP and D-dimer were measured before and every month after treatment during therapy. The rates of change of AFP and D-dimer were calculated as (AFP−AFP_baseline_)/AFP_baseline_ and (D-dimer−D-dimer_baseline_)/D-dimer_baseline_. The tumor and PVTT diameters were measured before and every month after treatment using contrast-enhanced CT. Tumor diameter and PVTT were defined as those areas of the tumor showing arterial enhancement on contrast-enhanced CT. The rates of change rate of tumor and PVTT diameters were calculated as (tumor diameter−tumor diameter_baseline_)/tumor diameter_baseline_ and (PVTT−PVTT_baseline_)/PVTT_baseline._

Liver and kidney functions and serum HBV DNA were measured pre- and post-treatment, and the impacts of the combination therapy on these parameters were evaluated. Adverse events were assessed and recorded according to the Common Terminology Criteria (version 3.0) for adverse events [46].

### Statistical analyses

All statistical analyses were performed using the Statistical Package for Social Sciences (SPSS 19.0 for Windows; SPSS Inc., Chicago, IL, USA) and Prism 7 (GraphPad Software, San Diego, CA, USA). Life tables and Kaplan–Meier survival curves were used to estimate overall and progression-free survival. The mean of all the patients’ data was used as the cut-off value for analysis of the clinical impact of treatment. Measured and numerical data were compared using *t*-tests and χ^2^ tests, respectively. Correlations between two variables were analyzed by linear regression. Variables with *P* < 0.05 were defined as statistically significant.
